# The characteristics of chronic benzene poisoning in 176 Chinese occupational population cases

**DOI:** 10.3389/fpubh.2024.1498114

**Published:** 2025-01-20

**Authors:** Lian Gou, Xingyu Ma, Lili Huang, Mei Qiu, Ruiqing Guo, Jun Jia, Peiyu Xu, Nan Lian

**Affiliations:** ^1^Department of Radiology, Huaxi MR Research Center (HMRRC), West China Hospital of Sichuan University, Chengdu, China; ^2^Sichuan Provincial Center for Mental Health, Sichuan Provincial People’s Hospital, School of Medicine, University of Electronic Science and Technology, Chengdu, China; ^3^Department of Nutrition, Food Hygiene, and Toxicology, West China School of Public Health, Sichuan University, Chengdu, China; ^4^Department of Health Promotion, Chongqing Traditional Chinese Medicine Hospital, Chongqing, China; ^5^Department of Animal Experiment, Sichuan Kelun Drug Research Institute Co., Ltd., Chengdu, China

**Keywords:** benzene poisoning, occupational health, exposure assessment, distribution, trend

## Abstract

Benzene is a widespread environmental carcinogen known to induce leukemia. Chronic benzene poisoning is a significant occupational health issue in China, particularly among workers exposed to benzene. The aim of this study was to analyze the distribution patterns and trends of occupational benzene poisoning cases. This study included 176 cases who are diagnosed with occupational chronic benzene poisoning, via the Occupational Disease Direct Network Reporting System of the Sichuan Center for Disease Control and Prevention from 2005 to 2019. Data on gender, date of birth, years of benzene exposure, enterprise size, ownership type, industry were collected and descriptively analyzed. No significant differences were observed between males and females in terms of age or benzene exposure duration. The variation in gender distribution across 4 periods highlighted significant differences (*χ*^2^ = 13.06, *p* = 0.004). Linear regression analysis indicated that the number of workers increased with year as the independent variable (*r^2^* = 0.40, *p* = 0.016). The working duration of benzene exposure appeared to decline, but this trend was not statistically significant. The majority of employees were in medium and large-sized enterprises. Before 2016, workers were mainly in joint-stock enterprises and equipment manufacturing industries; however, from 2017 to 2019, benzene poisoning cases were increasingly found in private and light industries. Overall, this study may provide data resources for risk assessment among occupational benzene-exposed workers; therefore, the monitoring of benzene concentrations in the workplace should be strengthened, and targeted preventive measures for workers must be effectively implemented to protect their health.

## Introduction

Benzene, the simplest organic aromatic hydrocarbon, is a common organic solvent used in various industrial, commercial and research projects, including plastics, resins, lubricants, rubbers, dyes, detergents, gasoline, and drugs (1). Global Benzene demand stood at 52.94 million tonnes in 2020 and is forecast to reach 78.03 million tonnes by 2030 (2). Owing to rapid economic and industrial development, benzene consumption and production in China have surged at a much higher average rate than any other region, making it a major consumer with imports worth USD 2.785 billion in 2021 (3). The increase is attributed to the growing benzene use in construction and automobile products.

High levels of benzene exposure typically occur in occupational settings, particularly through inhalation in industries such as rubber production, oil refining, packaging, and electronics manufacturing (4, 5). From 2001 to 2016, the mass concentration of benzene ranges from 0.19 to 53.90 μg/m^3^, with a mean of 6.48 ± 7.19 μg/m^3^, which is significantly lower than China’s indoor air quality standard (110 μg/m^3^,1-h average, GB/T 1883–2002) but higher than the guideline values of Japan, New Zealand, and the European Union (6). The permissible concentration-time weighted average (PC-TWA) of benzene in workplace in China is 6 mg/m^3^ (GBZ 2.1–2019), which is also higher than the U.S. limit of 3.25 mg/m^3^ (7).

The International Agency for Research on Cancer (IARC) has classified benzene as a group 1 human carcinogen (8). Epidemiological and toxicological evidence supports its carcinogenicity in occupational and non-occupational settings, and is associated with an increased risk of lung, colorectal, and bladder cancer (9–11). Benzene exposure also induces reproductive dysfunction, gut microbiota dysbiosis, and metabolic disorder (12, 13). The Occupational Safety and Health Administration (OSHA) limits benzene exposure in the air of most workplaces to 1 part per million (ppm) (14). Acute exposure to high doses of benzene has a negative impact on the nervous system, leading to dizziness, headaches, tremors, confusion, and/or unconsciousness (15, 16). Chronic long-term exposure to benzene in ambient air primarily harms the hematopoietic system (17, 18). Occupational exposure to benzene is associated with leukemia, particularly acute myeloid leukemia (AML), with increasing global morbidity and mortality (19, 20). Even less than 1 ppm (3.25 mg/m^3^) of ambient benzene also can cause non-carcinogenic hematotoxicity effects (21); workers do not show significant white blood cell (WBC) damage, but their bone marrow’s hematopoietic capability for renewal and differentiation is severely reduced (22).

Occupational chronic benzene poisoning has a long disease course, and there is no effective treatment for systemic damage. Retrospective exposure assessment is challenging but essential for detecting relevant exposure-disease associations. In this study, we investigated 176 benzene poisoning workers in Sichuan from 2005 to 2019 and conducted a descriptive analysis of their characteristics. This research may provide valuable data resources for risk assessment, safety evaluation and the establishment of occupational benzene exposure limits. For occupational benzene-exposed workers, once identified and diagnosed, timely and effective treatment can be obtained at an early stage.

## Methods

### Data source and processing

Data on 176 benzene poisoning cases between 2005 and 2019 were obtained from the Occupational Disease Direct Network Reporting System of the Sichuan Center for Disease Control and Prevention. Data included gender, date of birth, working duration of benzene exposure, enterprise size, ownership type, industry. The size of enterprises was classified according to the “Measures for the Classification of Large, Medium, Small, and Mini-sized Enterprises in Statistics (2017).” Based on these guidelines, enterprises were categorized into eight types: state-owned, collective, joint, private, foreign, direct-investment from Hong Kong, Macao and Taiwan, joint-stock and other economies. The division of industries was carried out by the “Industrial Classification for National Economic Activities (GB/T4754-2017).” The cases from various enterprises in each period were summarized. Moreover, we analyzed the distribution characteristics of the top 3 industries in each period from 2005 to 2009.

### Statistical analysis

Statistical analysis was performed with GraphPad Prism 8 and SPSS Statistics software. All data were presented as the mean ± SD for continuous variables or numbers (frequency and percentage) for categorical variables. Differences in age and years of benzene exposure between male and female workers were assessed using unpaired, two-tailed Student’s *t*-test. The differences in the number of male and female employees, enterprise scale, and ownership type across 4 periods were analyzed with Chi-square test. Linear regression analysis was conducted on the number of workers and working duration, with year as the independent variable. When *p* < 0.05, the difference was considered statistically significant.

## Results

### General characteristics of benzene poisoning workers

**Figure 1:**
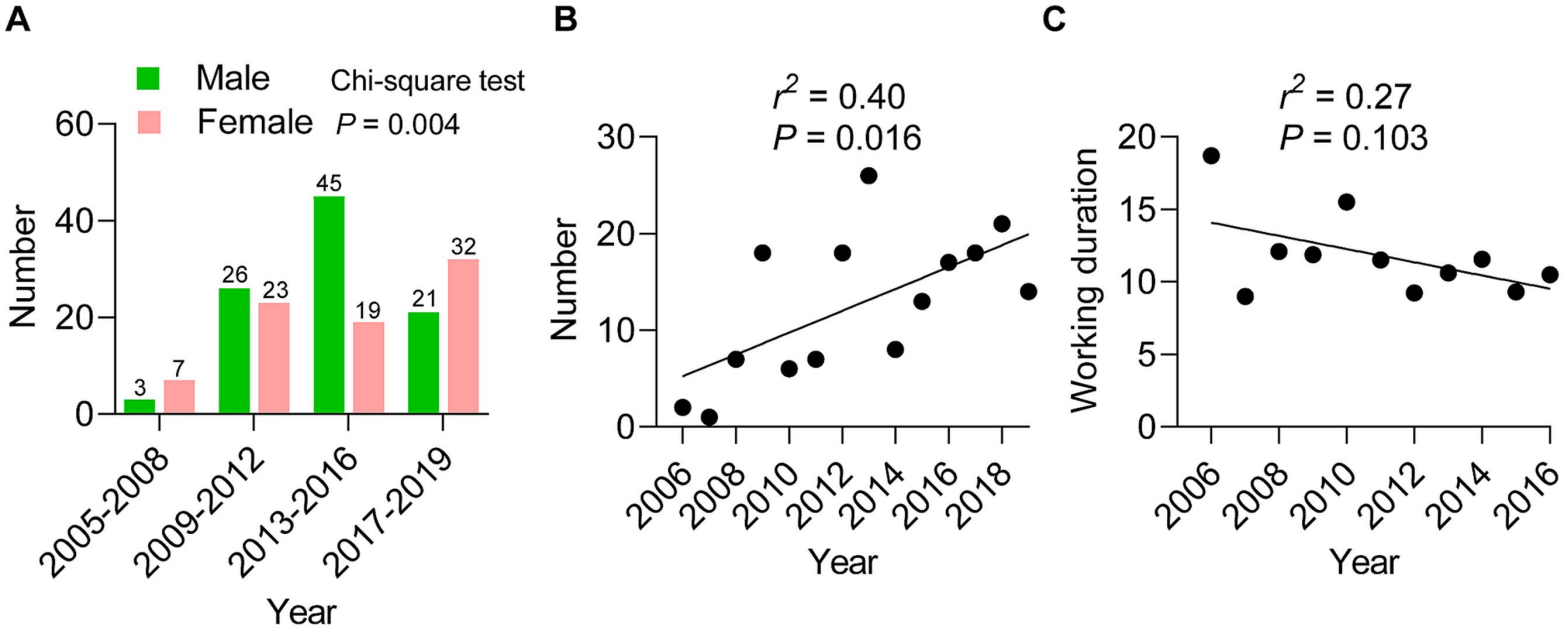
General characteristics of benzene poisoning workers in Sichuan, 2005–2019. **(A)** The number of male and female benzene poisoning cases across 4 periods (2005–2008, 2009–2012, 2013–2016, and 2017–2019); Chi-square test, *χ*^2^ = 13.06, *p* = 0.004. **(B)** The number of benzene poisoning cases from 2016 to 2019; linear regression analysis, *r*^2^ = 0.40, *p* = 0.016. **(C)** The working duration of these cases in 2006–2016, linear regression analysis, *r*^2^ = 0.27, *p* = 0.103.

A total of 176 benzene poisoning workers were recruited from 2005 to 2019, comprising 95 males (54.00%) and 81 females (46.00%). The average age of workers was 43.11 ± 9.24 years, and the ages of males and females were 42.73 ± 9.18 and 43.70 ± 9.39 years, respectively. The durations of exposure in males and females were 10.84 ± 7.79 and 11.24 ± 7.67 years, respectively, with an overall average of 11.00 ± 7.71 years. No significant differences were seen in age or benzene exposure duration (Table 1). We further analyzed the variation in gender distribution across 4 periods: 2005–2008, 2009–2012, 2013–2016, and 2017–2019. Intriguingly, the male population increased until 2016 and then dropped sharply; however, the female population continued to grow, indicating a difference in gender distribution across these periods (Figure 1A; *χ*^2^ = 13.06, *p* = 0.004). Linear regression analysis indicated that the number of workers increased with year as the independent variable (Figure 1B; *r*^2^ = 0.40, *p* = 0.016); while the working duration of benzene exposure appeared to decline, but this trend was not statistically significant (Figure 1C).

**Table 1:** The gender, age and years of benzene exposure in 176 cases.

Items	Number (%)	Age		Years of exposure	
		Means ± SDs	*P* value^a^	Means ± SDs	*P* value^a^
Male	95 (54.00)	42.73 ± 9.18	0.490	10.84 ± 7.79	0.732
Female	81 (46.00)	43.70 ± 9.39		11.24 ± 7.67	
		95% CI: 41.47–44.76		95% CI: 9.62–12.37	
Total	176 (100.00)	43.11 ± 9.24		11.00 ± 7.71	

^aUnpaired, two-tailed Student’s t-test.^

### The distribution and trends within enterprises and industries

The enterprise scale, ownership type and industry distribution of 176 benzene poisoning workers were further analyzed. As shown in Supplementary Table 1, these workers were mainly concentrated in medium-sized enterprises, with 68 of 176 cases (38.64%), followed by large enterprises with 51 cases (28.98%), and small enterprises with 38 cases (21.59%). Regarding ownership type, joint-stock economic enterprises accounted for the greatest number of cases, with nearly 73 cases (41.48%), followed by private enterprises with 48 cases (27.27%), and state-owned enterprises with 25 cases (14.20%). The above three types constituted approximately 83% of the total workers. In terms of industry distribution, more than 40% of cases were employed in the top 7 industries: transportation equipment manufacturing, general and professional equipment manufacturing, computer and other electronic equipment manufacturing, paper and cardboard container manufacturing, machinery for construction engineering, packaging services, weapons and ammunition manufacturing.

Next, we investigated the discrepancy in enterprise size and ownership type at each period. As shown in Table 2, benzene poisoning workers mainly occurred in medium and large-sized enterprises (*χ*^2^ = 22.62, *p* = 0.031), and the proportion increased from 50.00% (in 2005–2008) to 77.55% (in 2009–2012), decreased slightly to 54.69% (in 2013–2016), then increased to 77.36% (in 2017–2019). When enterprises were categorized according to the type of ownership, the number of cases varied significantly across each period (*χ*^2^ = 55.75, *p* < 0.001). The total number of workers in joint-stock enterprises was the highest from 2005 to 2016, then rapidly decreased to 28.30% in 2017–2019. The number of cases in private enterprises exhibited a steeper growth trend, reaching 54.72% in 2017–2019.

**Table 2:** Enterprise scale and ownership type in each period of 2005–2009.

Items	Number (%)			
	2005–2008 (*n* = 10)	2009–2012 (*n* = 49)	2013–2016 (*n* = 64)	2017–2019 (*n* = 53)
Enterprise scale^a^				
Mini-sized	0 (0.00%)	0 (0.00%)	1 (1.56%)	0 (0.00%)
Small	3 (30.00%)	4 (8.16%)	20 (31.25%)	11 (20.75%)
Medium	4 (40.00%)	25 (51.02%)	15 (23.44%)	24 (45.28%)
Large	1 (10.00%)	13 (26.53%)	20 (31.25%)	17 (32.08%)
Unknown	2 (20.00%)	7 (14.29%)	8 (12.50%)	1 (1.89%)
Ownership type^b^				
State-owned	1 (10.00%)	12 (24.49%)	5 (7.81%)	7 (13.21%)
Collective	0 (0.00%)	4 (8.16%)	6 (9.38%)	0 (0.00%)
Associates	0 (0.00%)	0 (0.00%)	5 (7.81%)	0 (0.00%)
Private	1 (10.00%)	4 (8.16%)	14 (21.88%)	29 (54.72%)
Foreign	2 (20.00%)	5 (10.20%)	5 (7.81%)	1 (1.89%)
Direct-investment from Hong Kong, Macao and Taiwan	0 (0.00%)	0 (0.00%)	0 (0.00%)	1 (1.89%)
Joint-stock	6 (60.00%)	24 (48.98%)	28 (43.75%)	15 (28.30%)
Others	0 (0.00%)	0 (0.00%)	1 (1.56%)	0 (0.00%)

^aChi-square test, χ2 = 22.62, p = 0.031. bChi-square test, χ2 = 55.75, p < 0.001.^

Moreover, we examined the distribution of top 3 industries with the most benzene poisoning cases in each period. As shown in Table 3, three industries covered all cases in 2005–2008. In 2009–2012, the workers were mainly distributed in transportation-equipment manufacturing (26.53% of the same period), weapon and ammunition manufacturing (16.33% of the same period), and general and professional equipment-manufacturing (12.24% of the same period). From 2013 to 2016, the ratio of workers in construction-machinery manufacturing ranked first, while the ratios in general and professional equipment-manufacturing and transportation-equipment manufacturing reduced. However, in 2017–2019, the distribution shifted toward the light industries, with 16.98% of cases in paper and cardboard container manufacturing and 15.09% in packaging service.

**Table 3:** Distribution characteristics in the top 3 industries in each period of 2005–2019.

Year	Top 3 industries	Number (%)
2005–2008	Computer and other electronic equipment-manufacturing	6 (60.00)
	General and professional equipment-manufacturing	3 (30.00)
	Chemical raw materials and chemical manufacturing	1 (10.00)
2009–2012	Transportation-equipment manufacturing	13 (26.53)
	Weapon and ammunition-manufacturing	8 (16.33)
	General and professional equipment-manufacturing	6 (12.24)
2013–2016	Construction-machinery manufacturing	9 (14.06)
	General and professional equipment-manufacturing	6 (9.38)
	Transportation-equipment manufacturing	4 (6.25)
2017–2019	Paper and cardboard-container manufacturing	9 (16.98)
	Packaging service	8 (15.09)
	Motor manufacturing	4 (7.55)

Overall, these results suggest that the majority of the 176 employees were in medium and large-sized enterprises; from 2005 to 2016, workers were predominantly in joint-stock enterprises and equipment manufacturing industries, while benzene poisoning cases were increasingly found in private and light industries in 2017–2019.

## Discussion

Human exposure to benzene has been associated with a range of acute and long-term adverse health effects and diseases, including cancer and hematotoxicity (23). In the present study, we investigated 176 benzene poisoning workers in Sichuan Province from 2005 to 2019. Our results demonstrated that the variation in gender distribution across 4 periods highlighted significant differences. The number of workers increased with year as the independent variable. The working duration of benzene exposure decreased, but this trend was not statistically significant. The majority of employees were in medium and large-sized enterprises. Before 2016, workers were mainly in joint-stock enterprises and equipment manufacturing industries; however, from 2017 to 2019, benzene poisoning cases were prevalent in private and light industries. This shift may be attributed to progressively stringent supervision and monitoring of enterprise and occupational health administration. Consequently, these findings not only reveal the characteristics of benzene poisoning workers but also provide a basis for optimizing occupational health conditions.

Sex-related biological differences may result in distinct health effects in women and men when exposed to benzene (24). Among the 176 benzene poisoning cases, the number of male cases increased until 2016 and then dropped sharply; however, the number of female cases continued to grow. In fact, there is no consensus regarding sex differences in response to benzene exposure. Diana et al. (24) have reported that male mice and rats are more susceptible to genotoxic and hematotoxic effects of benzene than females. Conversely, in human studies, published findings indicate that women may face a greater risk for adverse health effects associated with benzene exposure (25, 26). The sex differences are likely due to benzene’s interference with the pathways of endogenous hormones, but exact molecular mechanisms remain elusive.

A series of countermeasures have been issued to control atmospheric benzene homologues in China since 2010. The levels of exposure in most workplaces and benzene poisoning rate have been gradually decreasing. By 2017, the rate of chronic benzene poisoning had dropped from 1.1 to 0.054% in 6 provinces, China (27, 28). However, in this study, the number of benzene poisoning cases increased with year as the independent variable from 2006 to 2019. The increase may be associated with regional industrial output. In Sichuan, the furniture and electronic manufacturing sectors have developed comprehensive and robust industrial chains and clusters, making industrial emissions the main sources of ambient benzene. It is suggested that local governments should adopt differentiated control strategies for airborne benzene in the workplace. Moreover, even at exposure concentrations lower than 1 ppm, long-term benzene exposure has genotoxicity (29). Therefore, the exposure duration is an important factor for workers, and minimizing it is crucial for protecting the health of the occupational population. Genetics, physical activity, cigarette smoking, alcohol consumption, and dietary habits also exert a substantial synergistic effect on benzene poisoning.

These data provide an important scientific basis for the further revision of occupational disease prevention strategies. Further work is needed to address the limitations of this study. First, the present retrospective study made it impossible to measure the benzene concentration in the workplace and calculate the incidence accurately. Second, benzene-exposed workers with no poisoning and contemporaneously healthy individuals are not considered as controls. Finally, laboratory data (such as routine complete blood counts and urinary metabolites) of benzene poisoning cases might be missing. Thus, further research should focus more on gathering experimental data to establish benzene exposure thresholds.

According to the results of this investigation, targeted preventive measures for benzene-exposed workers should be effectively implemented to protect their health. Replacing benzene with low-toxic or non-toxic substances and reducing exposure duration can help reduce benzene exposure levels for workers. Promoting the use of personal protective equipment and improving the employee health management system are proactive measures to raise workers’ awareness of self-protection. Regular physical examinations are crucial for workers employed in enterprises with benzene exposure to facilitate early detection, diagnosis, and control the development of malignant hematological diseases.

## Conclusion

In Sichuan, China, there is an upward trend in the number of individuals diagnosed with occupational chronic benzene poisoning from 2005 to 2019. The network monitoring and reporting system for occupational diseases should be continuously improved. Routine measures encompass strengthening the surveillance of benzene concentrations and exposure duration in the workplace, promoting the wearing of protective equipment, and enhancing employee safety and health management systems.

## Data Availability

The raw data supporting the conclusions of this article will be made available by the authors, without undue reservation.
