# Supplementary material for: The characteristics of chronic benzene poisoning in 176 Chinese occupational population cases
Source: Front Public Health. 2025 Jan 20;12:1498114. doi: 10.3389/fpubh.2024.1498114 (PMC11788283; doi:10.3389/fpubh.2024.1498114)
Supplement: Supplementary file 1 [file Table_1.docx]

**Supplementary table**

**The characteristics of chronic benzene poisoning in a Chinese occupational population**

Correspondence: Nan Lian

**Supplementary Table 1.** Scale, type, and industry distribution of benzene poisoning enterprises

| Items | Number (n) | Ratio (%) |
| --- | --- | --- |
| **Enterprise scales** | | |
| Large | 51 | 28.98 |
| Medium | 68 | 38.64 |
| Small | 38 | 21.59 |
| Mini-sized | 1 | 0.57 |
| Unknown | 18 | 10.23 |
| **Enterprise types** | | |
| State-owned | 25 | 14.20 |
| Collective | 10 | 5.68 |
| Joint | 5 | 2.84 |
| Private | 48 | 27.27 |
| Foreign | 13 | 7.39 |
| Direct-investment from Hong Kong, Macao and Taiwan | 1 | 0.57 |
| Joint-stock | 73 | 41.48 |
| Other | 1 | 0.57 |
| **Industry distributions** | | |
| Transportation equipment manufacturing | 17 | 9.66 |
| General and professional equipment manufacturing | 15 | 8.52 |
| Computer and other electronic equipment manufacturing | 10 | 5.68 |
| Paper and cardboard container manufacturing | 9 | 5.11 |
| Machinery for construction engineering | 9 | 5.11 |
| Packaging services | 8 | 4.55 |
| Weapon and Ammunition manufacturing | 8 | 4.55 |
| Other | 100 | 56.82 |
